# Descriptive analysis of oral food challenge outcomes at a tertiary care center

**DOI:** 10.1186/1710-1492-7-S2-A7

**Published:** 2011-11-14

**Authors:** Elissa M Abrams, Nestor Cisneros, Allan B Becker

**Affiliations:** 1 Department of Pediatrics and Child Health, University of Manitoba, Winnipeg, Manitoba, Canada

## Background

Oral food challenges are the gold standard for clinical tolerance. Predictors of failing a challenge are needed for clinicians.

## Methods

A retrospective chart review of 2010 food challenges was performed. Descriptive analysis follows.

## Results

We assessed 113 challenges (35 peanut, 28 egg, 12 tree nut, 11 milk, 27 other). There were 29 failures (22 objective, 7 subjective). Among objective challenge failures, 4/7 cashew (57%), 10/35 (29%) peanut, and 6/28 egg (21%) failed. There were no failed milk challenges. Most (79%) failed peanut/cashew challenges occurred at doses ≤1.0g while 50% of failed egg challenges were final dose (10g). Three children required epinephrine (all cashew), none of whom had a prior known exposure (skin tested 2° peanut/almond). For peanut failures, 40% were history negative. The remainder of the challenge failure reactions were similar to the presenting reaction. Factors for failed challenges compared with successful challenges included atopic dermatitis (100% v 75%), asthma (93% v 63%), and other food allergy (64% v 48%).

## Conclusions

The majority of challenge failures were to peanut while the most severe reactions were to cashew, and occurred in patients without prior known exposure. Failures to peanut and cashew occurred at low doses while most egg reactions occurred at high doses. Those who failed a challenge had more atopic disease than those who passed.

